# Integrated Analysis of Transcriptome and Small RNAome Reveals the Regulatory Network for Rapid Growth in *Mikania micrantha*

**DOI:** 10.3390/ijms231810596

**Published:** 2022-09-13

**Authors:** Xiaowei Mo, Haolang Chen, Xiaolan Yang, Beixin Mo, Lei Gao, Yu Yu

**Affiliations:** Guangdong Provincial Key Laboratory for Plant Epigenetics, Longhua Bioindustry and Innovation Research Institute, College of Life Sciences and Oceanography, Shenzhen University, Shenzhen 518060, China

**Keywords:** *Mikania micrantha*, rapid growth, transcriptome, small RNA, degradome, regulatory network

## Abstract

*M. micrantha* has caused huge ecological damage and economic losses worldwide due to its rapid growth and serious invasion. However, the underlying molecular mechanisms of its rapid growth and environmental adaption remain unclear. Here, we performed transcriptome and small RNA sequencing with five tissues of *M. micrantha* to dissect miRNA-mediated regulation in *M. micrantha*. WGCNA and GO enrichment analysis of transcriptome identified the gene association patterns and potential key regulatory genes for plant growth in each tissue. The genes highly correlated with leaf and stem tissues were mainly involved in the chlorophyll synthesis, response to auxin, the CAM pathway and other photosynthesis-related processes, which promoted the fast growth of *M. micrantha*. Importantly, we identified 350 conserved and 192 novel miRNAs, many of which displayed differential expression patterns among tissues. PsRNA target prediction analysis uncovered target genes of both conserved and novel miRNAs, including *GRFs* and *TCPs*, which were essential for plant growth and development. Further analysis revealed that miRNAs contributed to the regulation of tissue-specific gene expression in *M. micrantha*, such as mmi-miR396 and mmi-miR319. Taken together, our study uncovered the miRNA-mRNA regulatory networks and the potential vital roles of miRNAs in modulating the rapid growth of *M. micrantha*.

## 1. Introduction

*Mikania micrantha* (*M. micrantha*, belonging to the family Asteraceae) is native to tropical America and is listed as one of the 10 most invasive weeds in the world [1,2]. Due to its fast-growing vines, which can grow up to 20 cm in one night [3], this plant species is named “mile-a-minute” weed [4,5,6]. In China, *M. micrantha* is also known as the “plant killer”. The rapid elongation of the cane makes it climb to the top of other plants, prevent other plants from receiving enough sunlight, and hinder their growth and reproduction, which eventually leads to the death of other plants and the destruction of genetic and species diversity [4,7].

To figure out the factors that contribute to the rapid growth and notorious invasion of *M. micrantha*, scientists analyze the population genetics of this species to trace its origin and the invasion history by using several molecular markers [8,9,10]. Studies in China mainly focus on the *M. micrantha* grown in the southeastern region of Guangdong province and use ISSR (inter-simple sequence repeats) [11,12], SSR (simple sequence repeats) [8,9,13], AFLP (amplified fragment length polymorphisms) [14], and SNP (single nucleotide polymorphism) [10] markers to trace the origin of *M. micrantha*. In Mexico, Bravo-Monzon et al. analyzed the genetic structure of native Mikania populations with six SSR markers [15]. These analyses suggest that to the special sequences in *M. micrantha* genome may have significant effects on its growth, metabolism, and defense responses.

A few studies have begun to uncover the function of *M. micrantha* genes. Zhou et al. showed that ABA treatment, dehydration and high-salt conditions could induce the expression of a two-fingered C2H2-type *ZFP* gene (*MmZFP1*), which plays a key role in drought resistance [16]. By transcriptome analysis, Huang et al. showed that genes involved in genomic evolution, plasticity, secondary metabolism and defense response in *M. micrantha* have homologous sequences from existing proteins in the NCBI database [17]. Based on the homologous sequence alignment, the genes encoding chlorophyll a/b binding protein were selected as the targets of RNA interference molecules that silence the expression of endogenous genes, and it revealed that these genes were essential for the survival of *M. micrantha* [18]. In 2020, through the integrated analysis of whole-genome sequencing, transcriptome and metabolome of *M. micrantha*, Liu et al. identified a genome-wide duplication event and recent segmental duplications, which may contribute to its quick adaption to the environment. They also found that the metabolites of *M. micrantha* could improve the nitrogen utilization rate by enriching the microbes that participate in nitrogen cycling pathways. This plant species could absorb CO_2_ at night to further supplement the carbon fixation, making it obtain higher photosynthetic capacity and improve the photosynthetic efficiency of the stem [19].

Epigenetic regulation is another vital aspect in modulating plant growth and development, as well as adaption to environment. MicroRNAs (miRNAs) are a class of endogenous small RNAs (sRNAs) of about 20–24 nucleotides (nt) in length and regulate their target genes mainly at the post-transcriptional level [20]. Many miRNAs and their targets play very conserved roles at different growth and development stages of plants. For example, during the development of *Arabidopsis* taproots, miR163 targets *Paraxanthine Methyltransferase 1* (*PXMT1*) in a light-dependent manner at a suitable temperature to affect the root length [21]. The miR396 family not only regulates the target genes *growth-regulating factors* (GRFs) to affect the development of leaves, but also affects the length and cell cycle of cells in the root elongation zone by regulating the target gene *basic helix*–*loop*–*helix transcription factor 74* (*bHLH74*) [22,23]. Both miR858 and miR3954 regulate the flowering time. The overexpression of miR858 in *Arabidopsis* and overexpression of miR3954 in kumquat result in the early flowering of transgenic plants [24,25].

In plants, the sequences of miRNAs are completely or almost completely complementary to those of their target gene transcripts, and miRNAs repress the expression of target genes by cleaving the target transcripts or inhibiting the translation. The continuous development of high-throughput sequencing platforms and integrated analysis of small RNA and transcriptome enabled the large-scale identification of miRNAs and their target genes in diverse plant species, including non-model plants [26]. So far, a large number of miRNAs and their target genes have been identified in many plants, such as rice [27], wheat [28], sesame [29], cotton [30], and peanut [31]. However, the identification and functional study of miRNAs and the corresponding targets in *M. micrantha* are rarely reported. In this study, we performed small RNA-seq and RNA-seq with five tissues of *M. micrantha*, including root, stem, leaf, shoot apex, and flower. Hundreds of conserved and novel miRNAs were identified, many of which were also differentially expressed among the five tissues. Integrated analysis uncovered the miRNA-mRNA regulatory networks in *M. micrantha* and revealed the potential roles of miRNAs in modulating the rapid growth and development of *M. micrantha*, which will facilitate further research on functional genomics and effective control of the rapid growth of this species.

## 2. Results

### 2.1. Weighted Gene Co-Expression Network Analysis (WGCNA) of the Transcriptome

The transcriptome sequencing was performed with five tissues of *M. micrantha*. Three biological replicates were prepared for each tissue, and the results were highly producible (Figure S1). In total, around 428.47 million (M) raw reads were detected in 15 samples, and the reads aligned to the genome accounted for 70.15%, 81.24%, 77.47%, 71.38%, and 80.39% of raw reads in flower, leaf, root, shoot apex, and stem, respectively (Table S1).

**Figure 1:**
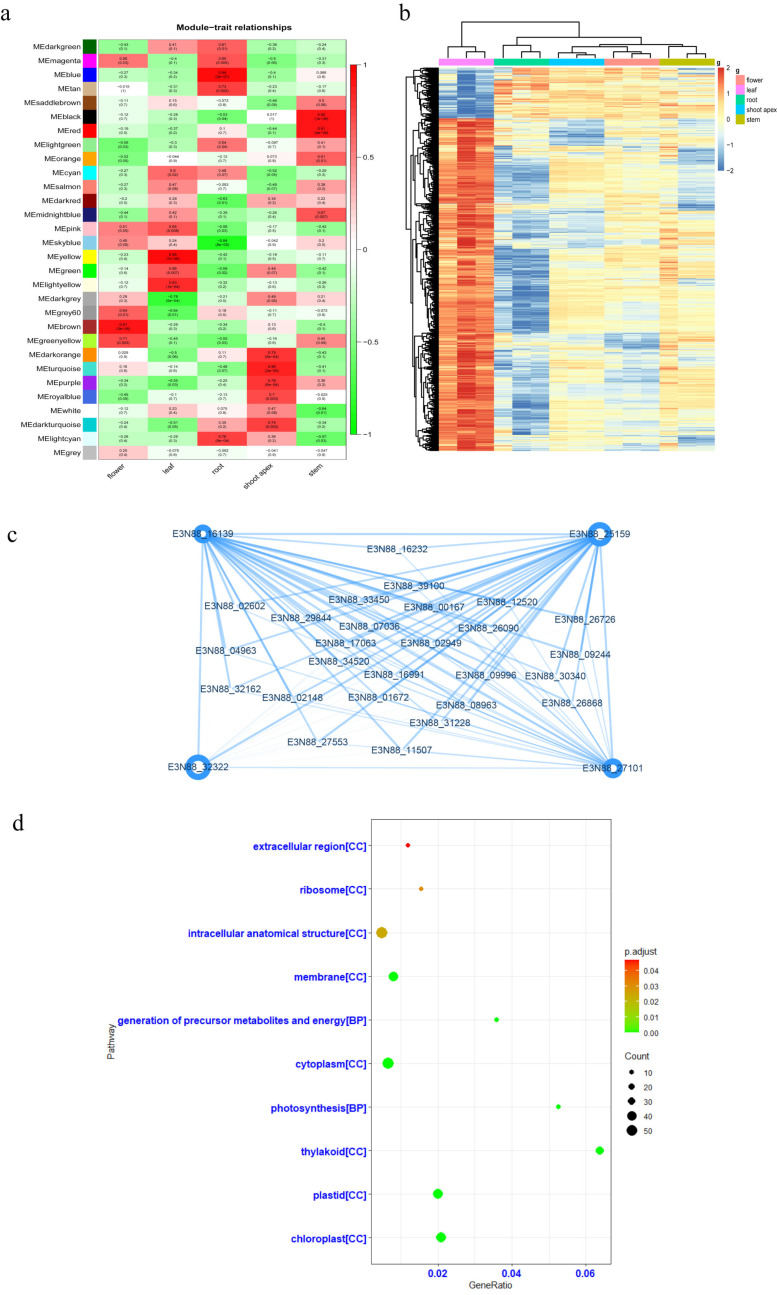
Co-expression module analysis of transcriptome in leaf tissue. (**a**) Module-tissue correlation analysis. The redder the color, the better the correlation. (**b**) Heatmap of yellow module genes in five tissues. Each tissue has three biological replicates. (**c**) Co-expression network of the top 30 genes with the highest correlation among leaf tissue genes. The size of the circle is represented by degrees, and the high and low correlation coefficients are represented by thick lines. (**d**) GO analysis of genes in the yellow module corelated to leaf tissue. GO analysis showed that genes related to photosynthesis and chloroplast pathways were enriched.

To understand the gene association patterns among different tissues and identify potential key regulatory genes for plant growth in each tissue, we carried out WGCNA with a total of 20,000 genes in all five tissues. As shown in Figure 1a, the correlation heat map was constructed by grouping the tissue types, and each gene module showed a significant correlation with one of the tissues, implying that there would be specific genes expressed in each tissue.

Most notably, the correlation coefficient between leaf tissue samples and the yellow-colored gene module reached 0.96, with the significance 7 × 10^−8^. Therefore, genes from the yellow module of leaf tissue were selected for the following analysis. First, 1721 genes in the yellow module were extracted to generate a grouped heatmap of gene expression (Figure 1b), and the expression level of these genes in leaves was significantly higher than that in other tissues. To explore the potential key regulatory genes in this module, we analyzed the co-expression network of the top 30 highly correlated genes (Figure 1c), with a correlation coefficient of 0.9 as the screening threshold. Four genes, E3N88_16139, E3N88_25159, E3N88_32322 and E3N88_27101, showed the highest correlation coefficients among the 30 genes, indicating that they may be the key genes in the yellow module of leaf tissue. Further analysis suggested that E3N88_16139 gene may encode a light-harvesting complex-like protein, E3N88_25159 gene may encode a photosystem light-harvesting complex -chlorophyll a/b binding protein, E3N88_32322 gene may encode a photosynthesis-related protein, and E3N88_27101 gene may encode a chloroplastic proton gradient regulation 5 (PGR5)-like protein 1A. In addition, GO (gene ontology) analysis of genes in the yellow module showed that these genes were mainly related to photosynthesis, metabolism and the generation of energy (Figure 1d). These results suggested that photosynthesis-related proteins are highly enriched in leaf tissue, and the above mentioned four genes could be the potential core genes responsible for the rapid growth of *M. micrantha*.

We also performed same co-expression analysis for other four tissues. For stem tissue, the black and red modules showed the highest correlation coefficients, in which eight genes were found to be the potential key genes, respectively (Figure S2). These key genes were mainly related to chlorophyll a/b binding, photosynthesis, response to auxin, and cell growth, which may explain the fast growth and elongation of the stems.

**Figure 2:**
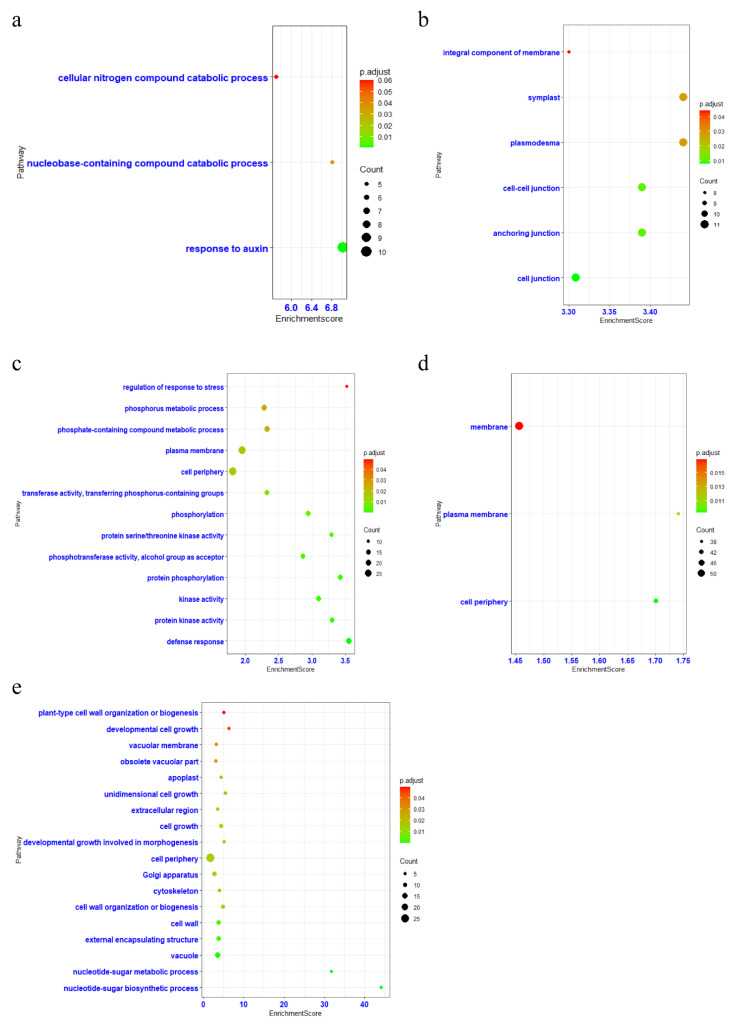
GO enrichment analysis of gene modules in each tissue. (**a**) GO analysis of genes in the black module correlated to stem tissue. (**b**) GO analysis of genes in the blue module correlated to root tissue. (**c**) GO analysis of genes in the light yellow module corelated to leaf tissue. (**d**) GO analysis of genes in the light cyan module correlated to root tissue. (**e**) GO analysis of genes in the red module correlated to stem tissue.

As for the flower, shoot apex, and root tissues, although some gene modules were correlated with each tissue, no specific key gene was identified. Therefore, we performed GO analysis on genes in the modules that correlated to each tissue (Figure 2). Similarly, the genes associated with leaf and stem tissues were mainly involved in chlorophyll synthesis, photosynthesis, response to auxin, cellular nitrogen compound catabolic process, defense response, developmental growth involved in morphogenesis and cell development, but few specific GO terms were enriched in the flower, root, and shoot apex tissues, respectively. Furthermore, several genes related to the CAM (crassulacean acid metabolism) pathway, mostly the carbon fixation process, were also identified in the leaf (E3N88_19214 and E3N88_33036, encoding the malate dehydrogenase (MDH) and carbonic anhydrase (CA), respectively), stem (E3N88_05396 and E3N88_05332, encoding MDH), and flower (E3N88_22852 and E3N88_22793, encoding malic enzyme (ME)) tissues, respectively (Figure S3), which were consistent with previous report that *M. micrantha* could fix CO_2_ through the CAM pathway [19]. To sum up, the rapid growth and development of *M. micrantha* could be mainly attributed to the genes related to photosynthesis and CAM pathways.

### 2.2. Small RNA Regulatory Pathways Exist in M. micrantha

**Figure 3:**
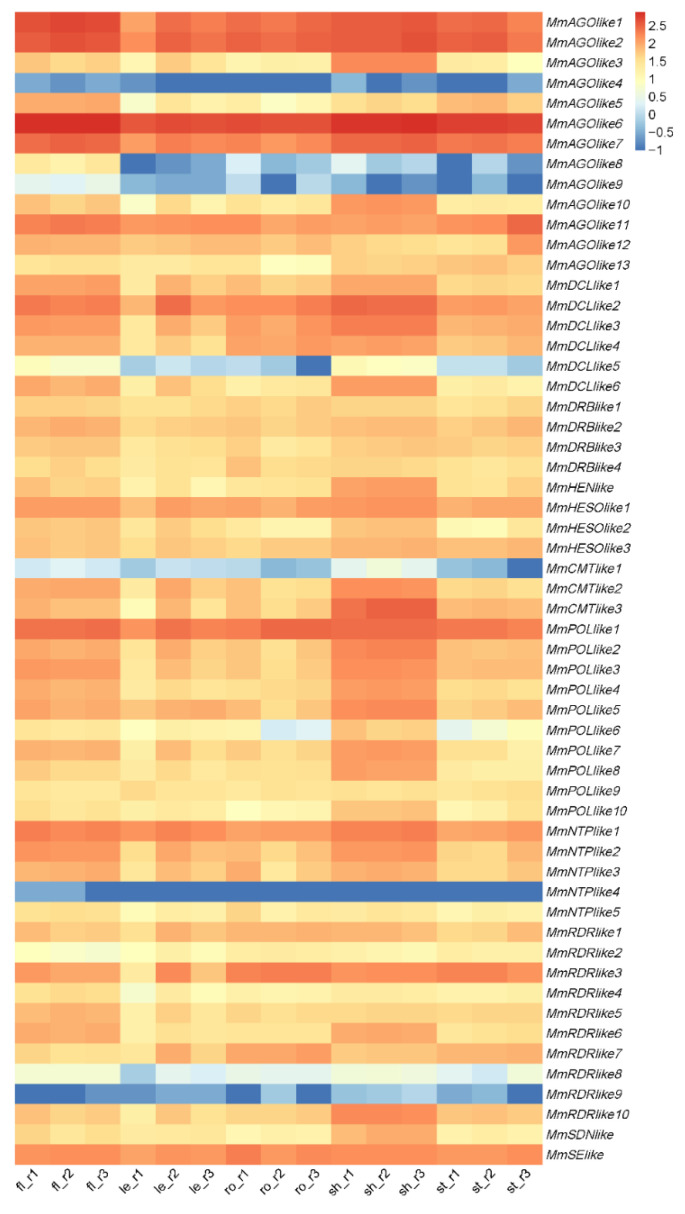
Expression of genes involved in sRNA biogenesis and regulatory pathways in five tissues of *M. micrantha*. fl, flower; le, leaf; ro, root; sh, shoot apex; st, stem. r1, r2, and r3 represent three biological replicates.

Besides coding genes, many long non-coding RNAs and small RNAs, especially miRNAs, were reported to play essential roles in plant growth and development. To determine whether small RNAs are produced in *M. micrantha* and contribute to the rapid growth and stress responses of this plant species, we first predicted the candidate genes that may participate in small RNA biogenesis and regulatory pathways by using HMMER as described [32,33], and analyzed their expression levels in the RNA-seq of five tissues. Several putative genes that may be involved in small RNA biogenesis in *M. micrantha* were identified, including 13 *Argonaute* (*AGOlike*), 6 *Dicer-like* (*DCLlike*), 4 *double-stranded RNA binding proteins* (*DRBlike*), 1 *HUA Enhancer 1* (*HENlike*), 10 *RNA-dependent RNA polymerases* (*RDRlike*), and 1 *Serrate* (*SElike*) (Figure 3). Some genes regulating sRNA degradation were also found, including 1 *small RNA Degrading Nuclease 1* (*SDNlike*), and 11 *Nucleotidyl Transferases* (3 *HESOlike* and 8 *NTPlike*). In addition, 3 *DNA methyltransferases* (*CMTlike*) were predicted, which could regulate RNA-directed DNA methylation (RdDM), suggesting that the RdDM pathway may present in *M. micrantha*. A total of 10 putative *RNA polymerases* (*POLlike*) were found as well, among which plant-specific Pol IV and Pol V should be included. Most of these predicted genes were highly expressed in the five tissues, especially in flower and shoot apex tissues, indicating that sRNA regulatory pathways exist in *M. micrantha* and potentially modulate its growth and environment adaption.

### 2.3. Identification of Conserved and Novel miRNAs in M. micrantha

To explore the regulatory function of sRNAs in *M. micrantha*, we constructed sRNA libraries with RNAs extracted from the five tissues. Three biological replicates were prepared for each tissue. Around 414.38 M raw reads were obtained in the flower, leaf, root, shoot apex, and stem in total. After filtering low-quality reads and trimming adapters, approximately 320.3 M reads were obtained, and the reads aligned to the genome were 13.19, 17.95, 4.51, 22.68 and 22.78 M in the flower, leaf, root, shoot apex, and stem, respectively (Table S2). The length of sRNAs ranged from 18 to 30 nt, and major sRNAs were 21–24 nt in length (Figure S4).

**Figure 4:**
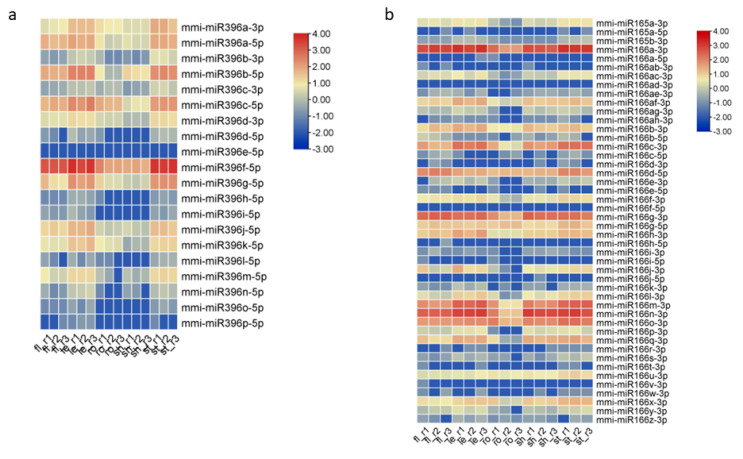
Expression of conserved miRNA families in the five tissues of *M. micrantha*. (**a**) Heatmap of mmi-miR396 family members in the five tissues. (**b**) Heatmap of mmi-miR165/166 family members in the five tissues. fl, flower; le, leaf; ro, root; sh, shoot apex; st, stem. r1, r2, and r3 represent three biological replicates.

With the published sRNA-seq database of diverse plant species, in total, 350 conserved miRNAs were identified from all five tissues of *M. micrantha*, among which mmi-miR396f-5p (UUCCACGGCUUUCUUGAACUG) was the most abundant one, with around 33,995 reads per million (RPM) (Table S3). When looking into each tissue, this miRNA also showed the highest abundance in flower, leaf, and stem. Other mmi-miR396 members were detected as well, and some were highly abundant in flower, leaf, and stem tissues (Figure 4a). Sequence comparison result revealed that there were only one to a few nucleotide differences among the mmi-miR396 family members. For example, the most abundant mmi-miR396f-5p differed from mmi-miR396a-5p (UUCCACAGCUUUCUUGAACUG) only in the 7th nucleotide and differed from mmi-miR396b-5p (UUCCACAGCUUUCUUGAACUU) in the 7th and 21st nucleotides. It is worth noting that the nucleotide differences among the mmi-miR396 family members would not affect their binding to or cleavage of the same target transcripts, indicating that mmi-miR396 members in *M. micrantha* may regulate the same target genes. Besides mmi-miR396, the mmi-miR165/166 family turned out to be the largest one with up to 46 members (Figure 4b), and they were also highly accumulated in different tissues. Similar to miR165/166 members in *Arabidopsis*, maize, rice, and other plants, mmi-miR165/166 family members showed sequence differences mainly at the 3’ end, suggesting that they would also share same targets. In addition, mmi-miR156, mmi-miR159, mmi-miR167, and mmi-miR319 family members exhibited generally high expression levels among the conserved miRNAs. Considering their high abundance and large family size, we speculated that these several mmi-miRNA families would play more vital roles than other miRNAs in *M. micrantha*.

**Figure 5:**
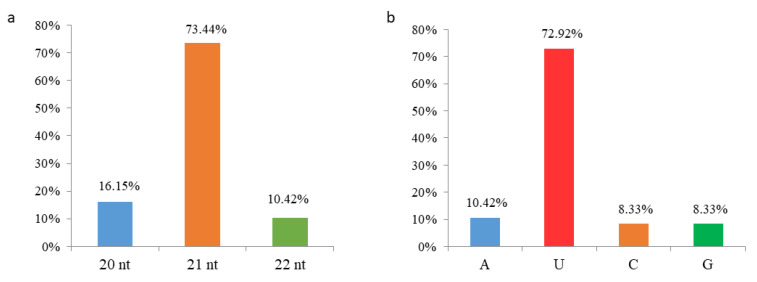
Characters of novel miRNAs in the five tissues in *M. micrantha*. (**a**) The length distribution of novel miRNAs. (**b**) Comparison of the 5’ first nucleotide of all novel miRNAs.

In addition to conserved miRNAs, we predicted novel miRNAs in five tissues via integrated analysis of sRNA-seq and RNA-seq according to previous described criteria [34]. A total of 192 novel miRNAs were identified from all five tissues (Table S4), and their precursors were able to form the stem-loop structure with several mismatches (Figure S5). These novel miRNAs were generally 20–22 nt in length, and 141 miRNAs (73.4%) were 21 nt (Figure 5a). Given that both the length and 5’ first nucleotide of miRNAs influence their associated AGO proteins and action modes, we analyzed the 5’ first nucleotide of all 192 novel miRNAs. A total of 140 novel miRNAs (72.9%) started with “U” at the 5’ end (Figure 5b), which was preferred by AGO1, indicating that the majority of novel miRNAs would be associated with AGO1 to mediate target transcript cleavage and/or translation repression. Among these novel miRNAs, the most abundant one, mmi-cand117 (UUAAAGCUUAGAAAAACGUCGU), with around 7511 reads per million (RPM), was highly expressed in all five tissues and started with “U”, implying its potential important roles via AGO1.

### 2.4. Differential Expression of miRNAs (DEmiRs) in Five Tissues of M. micrantha

In plants, many miRNAs exhibit specific temporal and/or spatial expression patterns, and their abundance is precisely and dynamically controlled via the balance between miRNA biogenesis and degradation pathways. For example, *Arabidopsis* miR172 is predominantly highly expressed in the floral organ to repress the translation of its target *APELA 2* (*AP2*), which is important for flowering [35]. In contrast, *Arabidopsis* miR163 is only abundant in the root and regulates the root length by targeting *PXMT1* [36].

**Figure 6:**
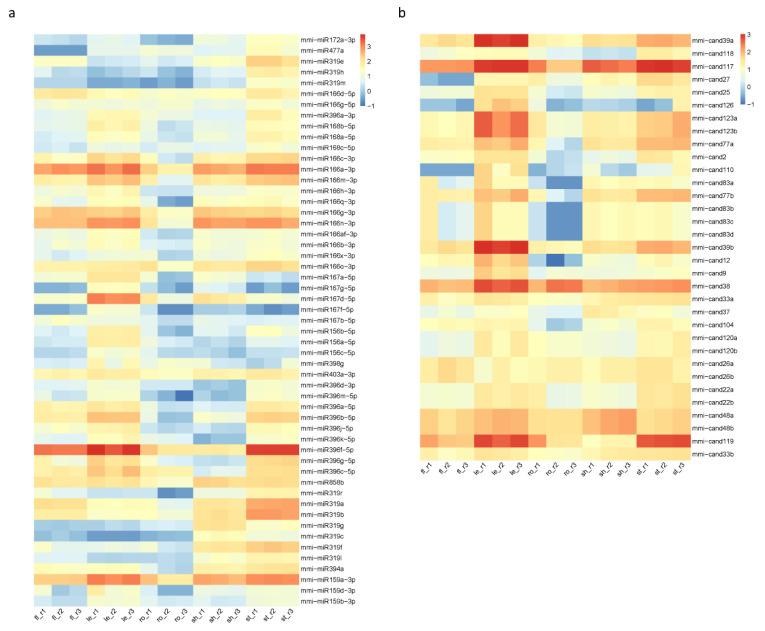
Differentially expressed miRNAs in the five tissues of *M. micrantha*. (**a**) Heatmap of differentially expressed conserved miRNAs in the five tissues. (**b**) Heatmap of differentially expressed novel miRNAs in the five tissues.

In order to verify whether *M. micrantha* miRNAs are also differentially expressed among different tissues, all identified conserved and novel miRNAs in the five tissues were compared with each other with the cutoff |log2(fold-change)| ≥ 1 and FDR ≤ 0.05. The analysis result showed that many mmi-miRNAs were differentially expressed in the five tissues (Figure 6). Among the conserved miRNAs, although the most abundant mmi-miR396f-5p were highly accumulated in flower, leaf, stem, and root tissues, its abundance in root and shoot apex was relatively lower (Figure 6a). Other mmi-miR396 family members also showed the similar expression pattern. We also performed Northern blotting to validate the differential expression of this miRNA family in the five tissues, and the result was consistent with sequencing analysis results (Figure S6). As for the largest family mmi-miR165/166, the members seemed to have different accumulation patterns among the five tissues. For example, mmi-miR166a-3p (UCGGACCAGGCUUCAUUCCCC) were highly abundant in all five tissues, mmi-miR166g-3p (UCGGACCAGGCUUCAUUCCUC) were enriched in flower and shoot apex, mmi-miR166d-5p (GGAAUGUUGUCUGGCUCGAGG) and mmi-miR166g-5p (GGAAUGUUGUUUGGCUCGAGG) were enriched in root tissue. Therefore, these miRNA family members did not show a significant difference in the abundance among the five tissues when detected by Northern blotting. In *Arabidopsis*, the miR165/166 family regulates leaf polarity and root xylem patterning by targeting the *class III homeodomain leucine-zipper* (*HD ZIP-III*) transcription factors [37,38], which were also conserved in other plant species. In tomato, the miR166-*SlHB15A* (belong to *HD ZIP-III* family) regulatory module controls ovule development and parthenocarpic fruit set under adverse temperatures [39]. In addition, the accumulation of mmi-miR319 family members in leaf tissue was generally lower than that in other tissues, which was confirmed by Northern blotting. The miR319 family participates in the regulation of leaf morphology and leaf senescence by modulating the *TEOSINTE BRANCHED 1* (*TB1*)/*CYCLOIDEA* (*CYC*)/*PROLIFERATING CELL NUCLEAR ANTIGEN FACTOR 1* (*PCF1*) (*TCP*) genes [40,41,42]. The overexpression of *TCP* genes or downregulation of miR319 results in delayed leaf senescence in *Arabidopsis* [40]. In tea plant *Camellia sinensis*, *CsTCPs*, targeted by CsmiR319b, regulate shoot tip development and catechin biosynthesis [43]. The differential expression of several other miRNAs, including mmi-miR159, mmi-miR167, mmi-miR168, and mmi-miR858, were also validated, and the results were consistent with sequencing analysis (Figure S6). Therefore, the differential expression of miRNAs, such as mmi-miR165/166 and mmi-miR319 family members, in the five tissues may contribute to the regulation of tissue-specific gene expression and the exuberant vitality of *M. micrantha*.

Novel miRNAs exhibited different spatial expression patterns as well (Figure 6b). For instance, mmi-cand117 (UUAAAGCUUAGAAAAACGUCGU) was highly expressed in all five tissues, and it was further enriched in the shoot apex and stem. The mmi-cand119 (UUCCACGGCUUUCUUGAACUA) was less abundant in the shoot apex than other four tissues. mmi-cand39a and mmi-cand39b (GGUAGUUCGACCGUGGAAUU) were most abundant in leaf tissue. In general, the differential expression of conserved and novel miRNAs suggested that numerous miRNAs would coordinately regulate the complicated biological processes in *M. micrantha*.

### 2.5. Prediction of miRNA Target Genes

To explore the regulatory functions of miRNAs identified in *M. micrantha*, we predicted their target genes using psRNA Target. With the standard of expectation set as 3, a total of 539 mmi-miRNAs corresponding to 4541 potential target genes were obtained, forming more than 12,000 miRNA-target pairs (Table S5). In this case, one miRNA may target multiple genes, such as the same gene family members, and one gene may be cleaved by more than one miRNA. For example, the most abundant mmi-miR396f-5p was predicted to target 52 genes, and the whole mmi-miR396 family members targeted 191 candidate genes, including the conserved *GRFs* genes that were involved in cotyledon and SAM development [44,45,46]. Novel targets were predicted for mmi-miR396, such as the genes encoding serine threonine-protein kinase and E3 ubiquitin-protein ligase, which might be involved in post-translation modification or protein degradation. The mmi-miR319 family had 18 members and putatively targeted 74 genes, many of which were *TCP* genes and responsible for leaf development. The largest mmi-miR165/166 family targeted around 272 genes, many of which belonged to the *HD ZIP-III* gene family regulating leaf and root development. The mmi-miR156 family consisted of 33 members and targeted 363 candidate genes. Most of these genes belonged to the *SPL* (*Squamosa promoter-binding-like protein*) gene family, which were conserved among plants and regulated the vegetative-productive phase transition. The mmi-miR858 family was predicted to target many *MYB* transcription factors which may participate in GA responses. Target genes for novel miRNAs were also predicted. In total, 192 novel miRNAs could target 1553 putative genes, but further validation of these miRNAs and their targets is required.

Surprisingly, we found that the mmi-miR845 family, consisting of mmi-miR845a/b/c, may target 585 genes. In *Arabidopsis*, miR845 is reported to target the long terminal repeat (LTR) retrotransposons in pollen to trigger the production of 21–22-nucleotide (nt) epigenetically activated small-interfering RNAs (easiRNAs) via Pol IV, and these easiRNAs are required for the triploid block [47]. In *M. micrantha*, the potential targets of mmi-miR845 included genes encoding ribosomal proteins, protein kinases, zinc finger proteins, histone methyltransferases, proteasome subunits and so on; many of these targets were related to gene expression regulation. Intriguingly, the gene encoding retrotransposon gag protein was also the putative target of mmi-miR845, suggesting that easiRNAs may be produced in *M. micrantha* pollen and required for post-fertilization genome stability and seed viability.

In addition, one target gene could be regulated by multiple miRNAs in *M. micrantha* (Table S6). For example, E3N88_38712, encoding RNA polymerases superfamily protein was predicted to be targeted by 139 mmi-miRNAs, including the mmi-miR165/166 family members, mmi-miR393d-5p and many novel miRNAs. E3N88_00987, encoding a mitochondrial protein, was regulated by 93 miRNAs, such as mmi-miR165/166 family members, mmi-miR845b and other novel miRNAs.

Although thousands of miRNA target genes were predicted in *M. micrantha*, many of which turned out to be the conserved target gene families among diverse plant species, further validation and functional investigation of these targets are needed to determine their roles in regulating the growth and environmental adaption of in *M. micrantha*.

### 2.6. Potential Roles of miRNAs in Regulating M. micrantha Development

mmi-miR396, mmi-miR165/166, mmi-miR319, and mmi-miR156 families were most abundant among the miRNAs, and their predicted conserved targets were required for the proper growth and development. To better understand the regulatory roles of these miRNAs in *M. micrantha*, we performed GO analysis for all predicted target genes of each family, respectively. Most of these genes were related to plant organ development and stress responses.

**Figure 7:**
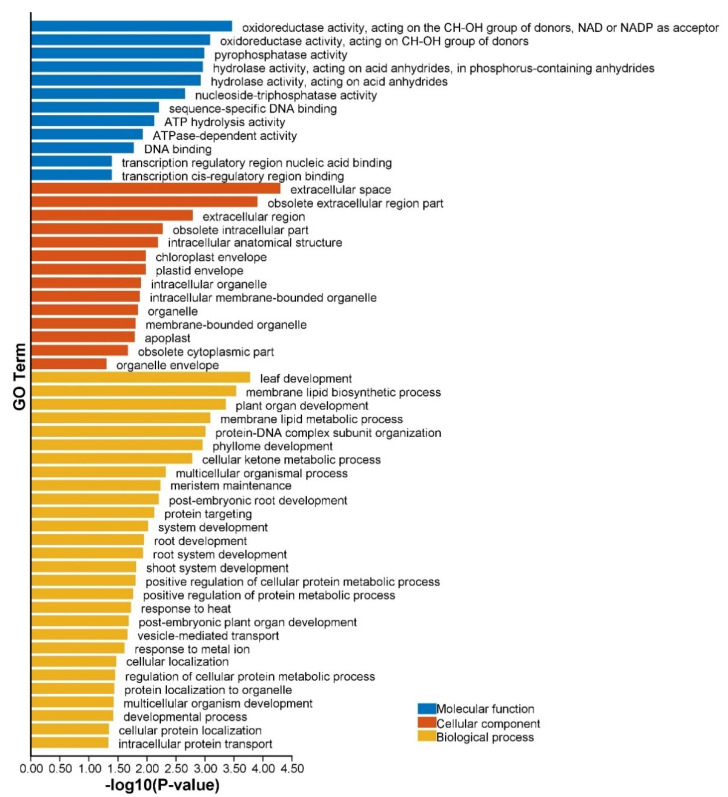
GO analysis of putative mmi-miR396 target genes.

The mmi-miR396 target genes were predominantly involved in organ development, such as leaf development, phyllome development, meristem maintenance, root development, shoot system development, multicellular organism development, and other developmental processes (Figure 7). The targets of mmi-miR165/166 participated in developmental processes, including establishment or maintenance of cell polarity, embryo development, and seed development (Figure S7). They also regulated many abiotic stress responses, such as response to nutrient levels and starvation. The targets of mmi-miR319 were majorly involved in leaf and floral organ morphogenesis and development, as well as responses to abiotic stresses, including response to salt stress, osmotic stress and other abiotic stimuli (Figure S8). The mmi-miR156 target genes were associated with leaf development, phase transition, shoot morphogenesis, floral organ development, meristem development, and many metabolic processes, such as nitrogen compound and organonitrogen compound metabolism (Figure S9). These results suggested that miRNA targets in *M. micrantha* should have played essential roles in modulating the proper development and environmental adaption of this species.

**Figure 8:**
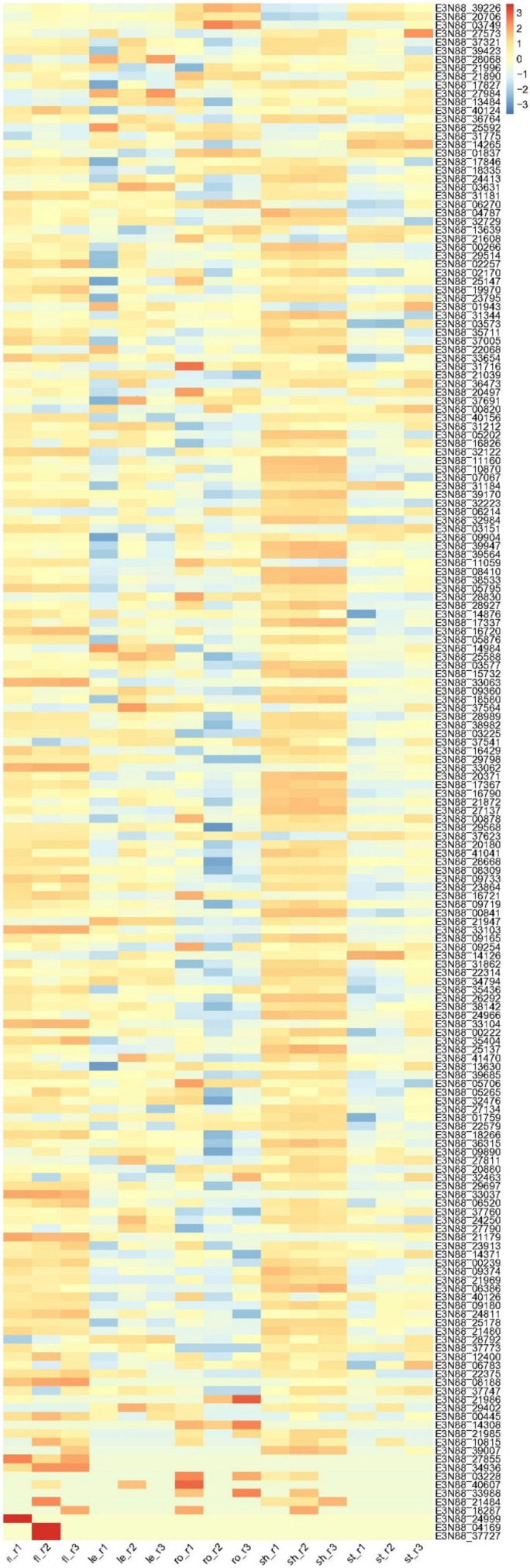
Expression of *GRF* genes putatively targeted by mmi-miR396 in five tissues of *M. micrantha*. fl, flower; le, leaf; ro, root; sh, shoot apex; st, stem. r1, r2, and r3 represent three biological replicates.

The differential expression patterns of miRNAs among the five tissues could be responsible for the regulation of tissue-specific gene expression, which may further influence the rapid growth of *M. micrantha*. For example, the abundance of mmi-miR396 members in shoot apex were relatively lower than those in the leaf and stem tissues, indicating that their target *GRF*s should accumulate at a higher level in the shoot apex. Indeed, in our transcriptome, the expression levels of putative *GRF* genes targeted by mmi-miR396 were higher in the shoot apex and flower than those in other tissues (Figure 8), which may facilitate the growth of SAM (shoot apical meristem), thereby promoting the rapid growth of *M. micrantha*. Mmi-miR319 members were less abundant in the leaf tissue, suggesting that the target *TCPs* would show a high expression level in leaves, which may delay the leaf senescence of *M. micrantha*. Taken together, the identified miRNAs and predicted targets helped to uncover the miRNA-mRNA regulatory network and indicate the essential roles of miRNA in the rapid growth of *M. micrantha*.

## 3. Discussion

As an invasive species, *M. micrantha* exhibits strong adaptability and rapid growth. It is reported that the fast growth of this species could be due to several strategies, such as improving the nitrogen utilization rate by enriching the microbes that participate in nitrogen cycling pathways and improving the photosynthetic efficiency by absorbing CO_2_ at night [19]. However, the underlying mechanisms are still unclear.

In this study, we conducted a comprehensive transcriptome and small RNAome analysis for *M. micrantha* to understand the regulatory network of miRNA-mRNA by which *M. micrantha* can grow fast and adapt to the environment. By WGCNA analysis of transcriptome, we explored the gene association patterns among different tissues of *M. micrantha* and identified the potential key genes that highly associated with each tissue, which may be responsible for the rapid growth of *M. micrantha*. For example, many key genes highly correlated with the leaf and stem tissues were involved in the photosynthesis processes and carbon fixation in the CAM pathway, indicating the larger photosynthetic area and the higher photosynthetic efficiency in *M. micrantha* than those in other species, which was in good accordance with previous report that *M. micrantha* absorbs CO_2_ at night and the stems also carry out photosynthesis [19]. The genes responding to auxin also showed high correlation coefficient to the stem, which may explain the fast elongation of canes.

In addition to the functional genes, miRNAs serve as the important epigenetic regulators of gene expression and majorly act at the post transcriptional level. The first miRNA was discovered in *C. elegans* in 1993 [48]; subsequently, miRNAs were identified in *Arabidopsis* in 2002 and the functions of miRNAs in plants have received extensive attention since then [49,50]. Numerous studies have uncovered the vital roles of miRNAs during plant growth and development in diverse plant species nowadays. By using HMMER, we predicted many homologues of small RNA pathways genes in *M. micrantha*, including the genes involved in small RNA biogenesis, degradation and the RdDM pathway. These genes were highly expressed in the five tissues, indicating that small RNA regulatory pathways exist in *M. micrantha* and may modulate the rapid growth of *M. micrantha*.

There were 350 conserved and 192 novel miRNAs identified in the five tissues of *M. micrantha*, and their targets were also predicted. Among the conserved miRNAs, mmi-miR396f-5p behaved with the highest abundance and was predicted to target many genes including the *GRFs*. mmi-miR165/166 possessed the largest family size, and mainly targeted the *HD ZIP-III* family. Many other miRNAs also accumulated at high levels, such as mmi-miR156, mmi-miR159 and mmi-miR319, which were predicted to target *SPLs*, *MYBs*, and *TCPs*, respectively. All of these target genes were also very conserved in plants and were required for the proper growth and development, suggesting the roles of conserved miRNAs in the rapid growth of *M. micrantha*. Novel target genes were also found for the conserved miRNAs, but their roles remained unknown. In addition, the majority of novel miRNAs were 21 nt in length and started with an “U” at the 5’ end, indicating that these novel miRNAs would inhibit the expression of target genes by transcript cleavage and/or translation repression through AGO1. The specific functions of these novel miRNAs and novel target genes should be investigated in the future.

Similar to other plants, the mmi-miRNAs exhibit differential expression patterns among different tissues, which could facilitate the regulation of tissue-specific gene expression and the exuberant vitality of *M. micrantha*. For example, the abundance of mmi-miR396 family in shoot apex was relatively low as compared to other tissues, indicating that their target *GRF* genes should have a relatively high expression level in shoot apex. In *Arabidopsis*, *Atgrf1/2/3/4* quadruple mutants displayed cup-shaped cotyledons, many mutants also lacked SAM [44,45,46], indicating that *AtGRFs* were required for the development of cotyledon and SAM. The aerial organs of vascular plants start from the SAM, and proper SAM growth and maintenance determine the growth of lateral organs (leaves) [51,52]. Therefore, the high expression of *GRFs* in the shoot apex tissue will facilitate the growth of SAM, thereby promoting the growth of leaves and the rapid growth of *M. micrantha*. Similarly, the abundance of the mmi-miR319 family was lower in *M. micrantha* leaf than in other tissues, implying that its target gene *TCPs* were highly expressed in leaf tissue. A previous study reported that miR319 was involved in the regulation of plant leaf morphology by targeting the *TCP2*, *TCP3*, *TCP4*, *TCP10*, and *TCP24* genes [41,42]. In addition, in *Arabidopsis*, the silence of miR319 or overexpression of *TCPs* caused delayed leaf senescence [40]. These results indicate that miR319 not only participates in the regulation of plant leaf morphology, but also has certain effects on plant leaf senescence. As the most important organ for photosynthesis, both the shape and senescence of leaves determine the photosynthesis efficiency. The high photosynthesis efficiency of leaves will promote the growth of plants. The low abundance of mmi-miR319 in *M. micrantha* leaf tissue will be beneficial to leaf morphology maintenance and senescence delay, enabling leaves to maintain a high photosynthetic efficiency, thereby promoting the rapid growth of *M. micrantha*. Taken together, these results indicate that miR396 and miR319 play important roles in regulating the growth and development of *M. micrantha*.

## 4. Materials and Methods

### 4.1. Plant Materials

Wild-type *M. micrantha* vines were collected from Shenzhen University (Lihu Campus) and cut into several branches with two stem nodes and a leaf on the apical side. Then the morphological base end was cultured in water under low light conditions for approximately 1 week. Plants with root lengths of approximately 4–5 cm were transferred to Hoagland liquid medium and grown in the growth room with daily cycles of 16 h/light and 8 h/dark at 24 °C for 60 days. The flower, leaf, shoot apex, stem, and root tissues were collected for RNA extraction; three biological repeats were prepared for each tissue.

### 4.2. RNA Extraction, Library Construction and Sequencing

Total RNA was extracted from *M. micrantha* flower, leaf, shoot apex, stem, and root tissues using TRIzol (Invitrogen, Carlsbad, CA, USA) according to the manufacturer’s instructions. The RNA integrity and concentration were determined with NanoDrop (Thermo Fisher, San Jose, CA, USA). The small RNA libraries were constructed as described before [53] and sequenced on the Illumina HiSeq 2500 platform with the 75SE sequencing strategy at Berry Genomics Co., Ltd. (Beijing, China). Transcriptome libraries were constructed and sequenced by Berry Genomics Co., Ltd. (Beijing, China).

### 4.3. WGCNA of Transcriptome

WGCNA analysis was performed as described before [54,55]. First, a scale-free distribution topology matrix was calculated to determine the optimal beta value, with the expression levels of all co-annotated genes in the 15 samples (five tissues, three biological replicates per tissue) as the input data, and power = 16 was chosen as the beta value for the subsequent analysis. Then a co-expression matrix of genes co-annotated with *Arabidopsis* was constructed, dividing the 20,000 genes into 30 modules. To specifically quantify the unique correlations between individual gene modules and each sample, the correlation coefficients of gene clustering modules and sample features were analyzed. Use plot Eigengene Networks to create modular cluster plots and modular heatmaps. Extract the genes in each module and use pheatmap to draw the gene expression heat map of the module and add tissue type grouping information. Finally, the correlation coefficient matrix between genes was imported into cytoscape as interaction information to create a gene co-expression network.

### 4.4. Identification and Validation of Differential Expression Analysis of Conserved and Novel miRNAs

The raw data were processed and mapped to the *M. micrantha* reference genome (ASM936387v1) by AUSPP with the mode (-M smallRNA) [19,56]. All remained reads were matched with plant miRNA dataset in miRBase release 22.1 [57] (http://www.miRBase.org/, accessed on 7 December 2021), and the matched reads were defined as conserved miRNAs. The potential novel miRNAs were predicted using miRDeep-P2 software (version 1.1.4, Xiaozeng Yang, Beijing, China) [58]. The normalized abundance of all miRNAs was displayed as RPM (reads per million mapped reads), and statistically compared among different samples using edgeR [59] with |log2(fold-change)| ≥ 1 and FDR ≤ 0.05. The differential expression of miRNAs among five tissues were validated by Northern blotting as described before [53]. The sequences of probes used in this study are listed in Table S7.

### 4.5. Prediction of miRNA Target Genes

miRNA target genes were predicted using the psRNATarget program [60] (https://www.zhaolab.org/psRNATarget/, accessed on 7 December 2021) with a maximum expectation of 3.

## 5. Conclusions

We performed co-expression network analysis of RNA-seq from five tissues to explore many candidate genes responsible for the rapid growth and development of *M. micrantha*. We also identified numerous conserved and novel miRNAs in *M. micrantha* and predicted the targets of mmi-miRNAs. According to the abundance and family size, mmi-miR396, mmi-miR165/166, and mmi-miR319 families may play the most important roles in regulating the growth and development of *M. micrantha* by targeting *GRF* genes, *HD ZIPIII* genes, and *TCP* genes, respectively. In addition, the differential repression of miRNAs among the five tissues may contribute to the regulation of tissue-specific gene expression and facilitate the fast growth of *M. micrantha*. Our results not only revealed a miRNA-mRNA regulatory network in *M. micrantha*, but also indicated the potential roles of miRNAs and their target genes in the fast growth and development of *M. micrantha*. These findings will provide new insights into dissecting the molecular mechanism of the rapid growth of *M. micrantha* and controlling the serious invasion of these invasive plants.

## Data Availability

All the data are shown in the main manuscript and in the Supplementary Materials. The sequencing data described in this manuscript were submitted to the National Center for Biotechnology Information (NCBI) under accession codes GSE210300.

## Supplementary Materials

The following supporting information can be downloaded at: https://www.mdpi.com/article/10.3390/ijms231810596/s1.
