# Supplementary material for: Integrated Analysis of Transcriptome and Small RNAome Reveals the Regulatory Network for Rapid Growth in Mikania micrantha
Source: Int J Mol Sci. 2022 Sep 13;23(18):10596. doi: 10.3390/ijms231810596 (PMC9501215; doi:10.3390/ijms231810596)
Supplement: Supplementary file 1 [file ijms-23-10596-s001.zip › Supplementary Materials.pdf]

Supplementary Materials

Figure S1. The correlation heatmap among biological replicates of RNA-seq from five tissues.

Figure S2. Co-expression network of the top 30 genes in the black (a) and red (b) modules with the highest correlation to stem tissue.

Figure S3. Expression pattern of genes related to the CAM pathway in five tissues.

Figure S4. Length distribution of small RNAs detected in the five tissues of *M. micrantha*.

Figure S5. Stem-loop structure of predicted novel miRNA precursors.

Figure S6. Validation of miRNAs in five tissues.

Figure S7. GO analysis of putative mmi-miR165/166 target genes.

Figure S8. GO analysis of putative mmi-miR319 target genes.

Figure S9. GO analysis of putative mmi-miR156 target genes.

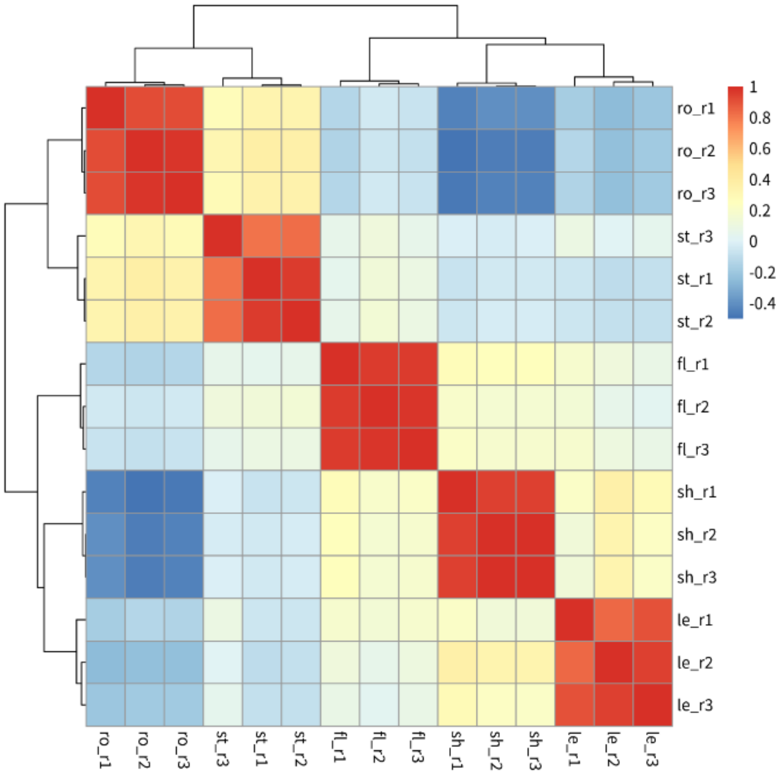

Figure S1. The correlation heatmap among biological replicates of RNA-seq from five tissues. fl, flower; le, leaf; ro, root; sh, shoot apex; st, stem. r1, r2, r3 represent three biological replicates.

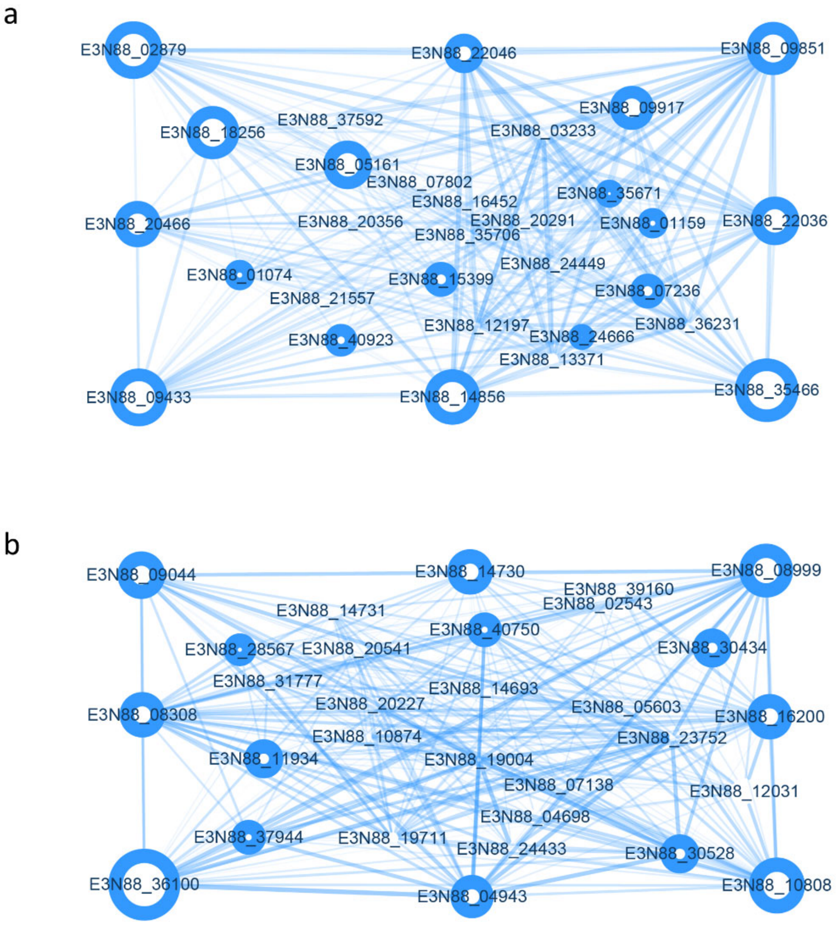

Figure S2. Co-expression network of the top 30 genes in the black (a) and red (b) modules with the highest correlation to stem tissue.

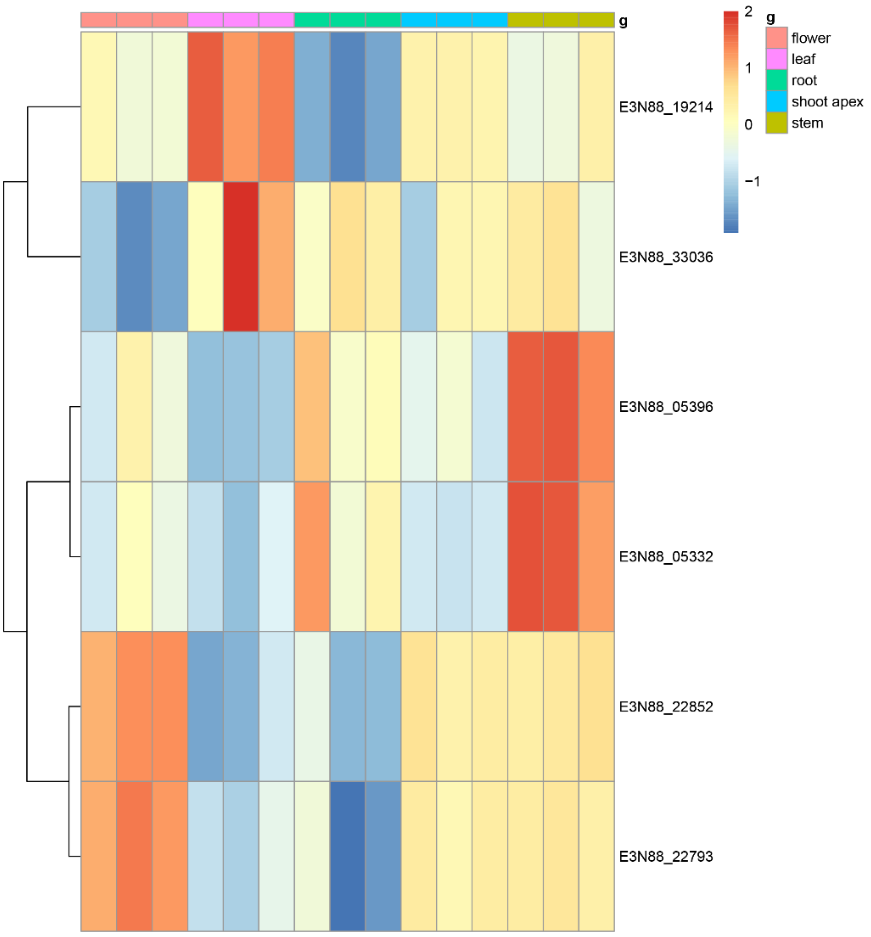

Figure S3. Expression pattern of genes related to the CAM pathway in five tissues.

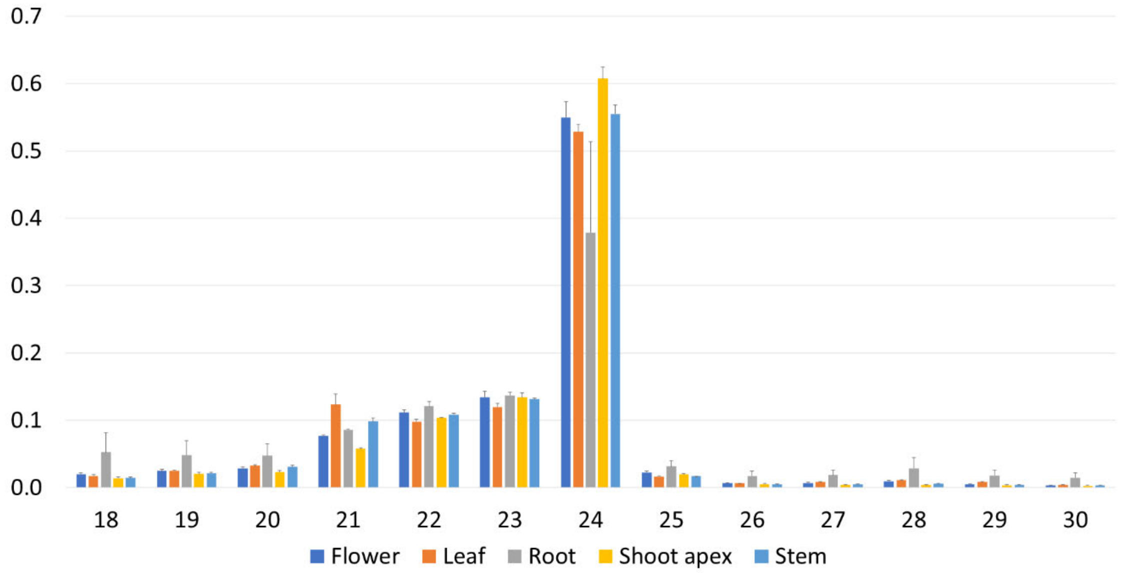

Figure S4. Length distribution of small RNAs detected in the five tissues of *M. micrantha*. X-axis represents the length (nt), Y-axis represents the relative percentage of sRNAs in each tissue, which is calculated based on the three biological replicates. Error bar represents standard deviation.

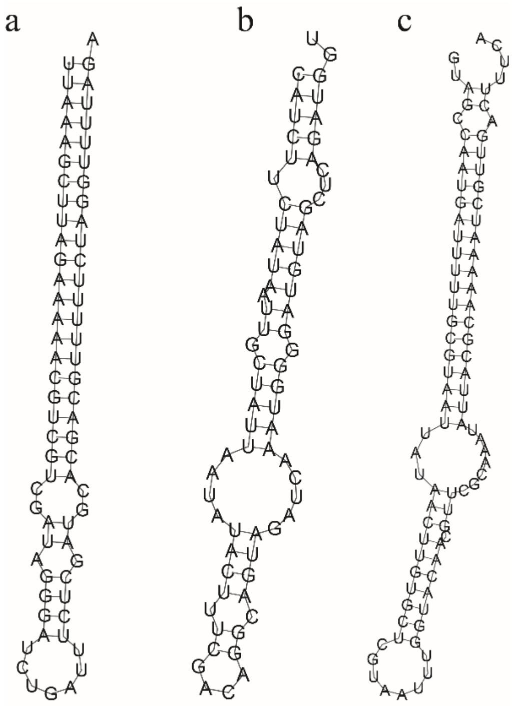

Figure S5. Stem-loop structure of predicted novel miRNA precursors. (a) Structure of mmi-cand117 precursor. (**b)** Structure of mmi-cand38 precursor. (**c)** Structure of mmi-cand26b precursor.

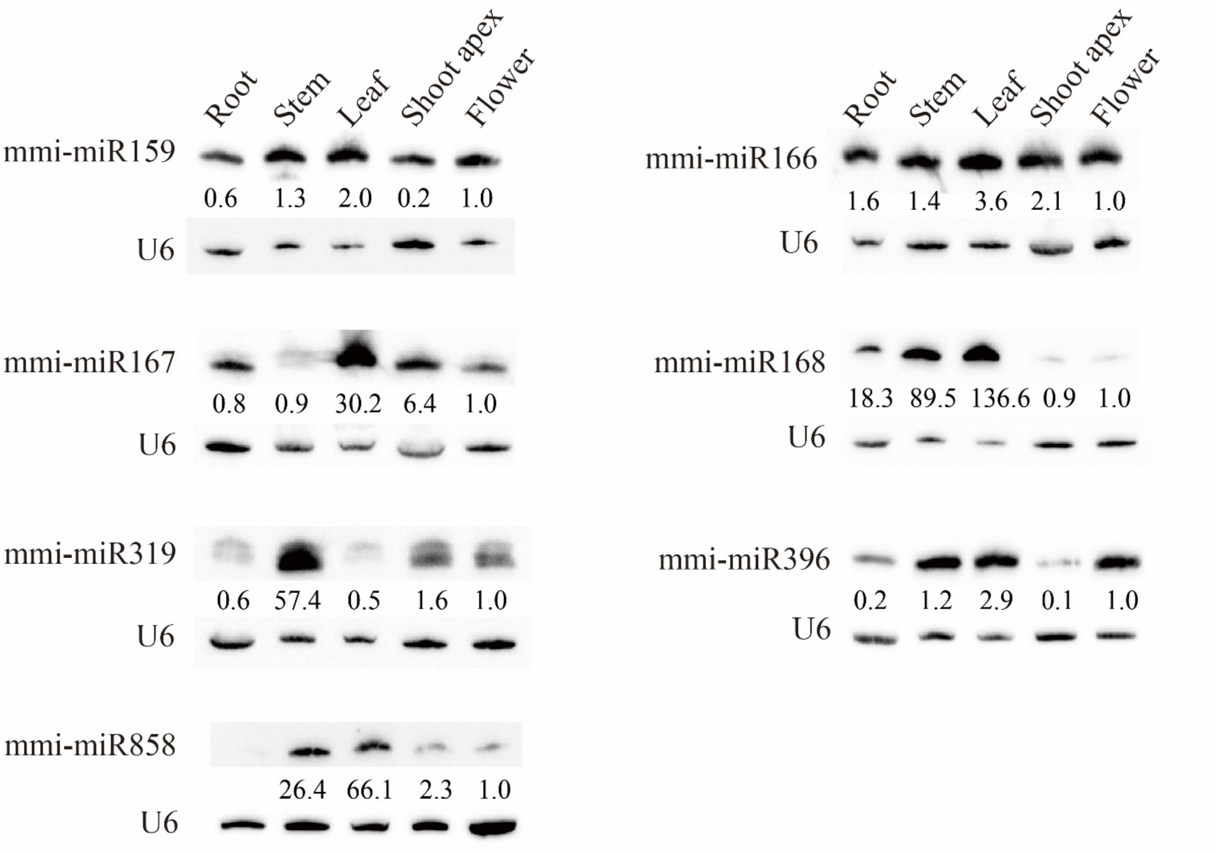

Figure S6. Validation of miRNAs in five tissues. U6 served as a loading control.

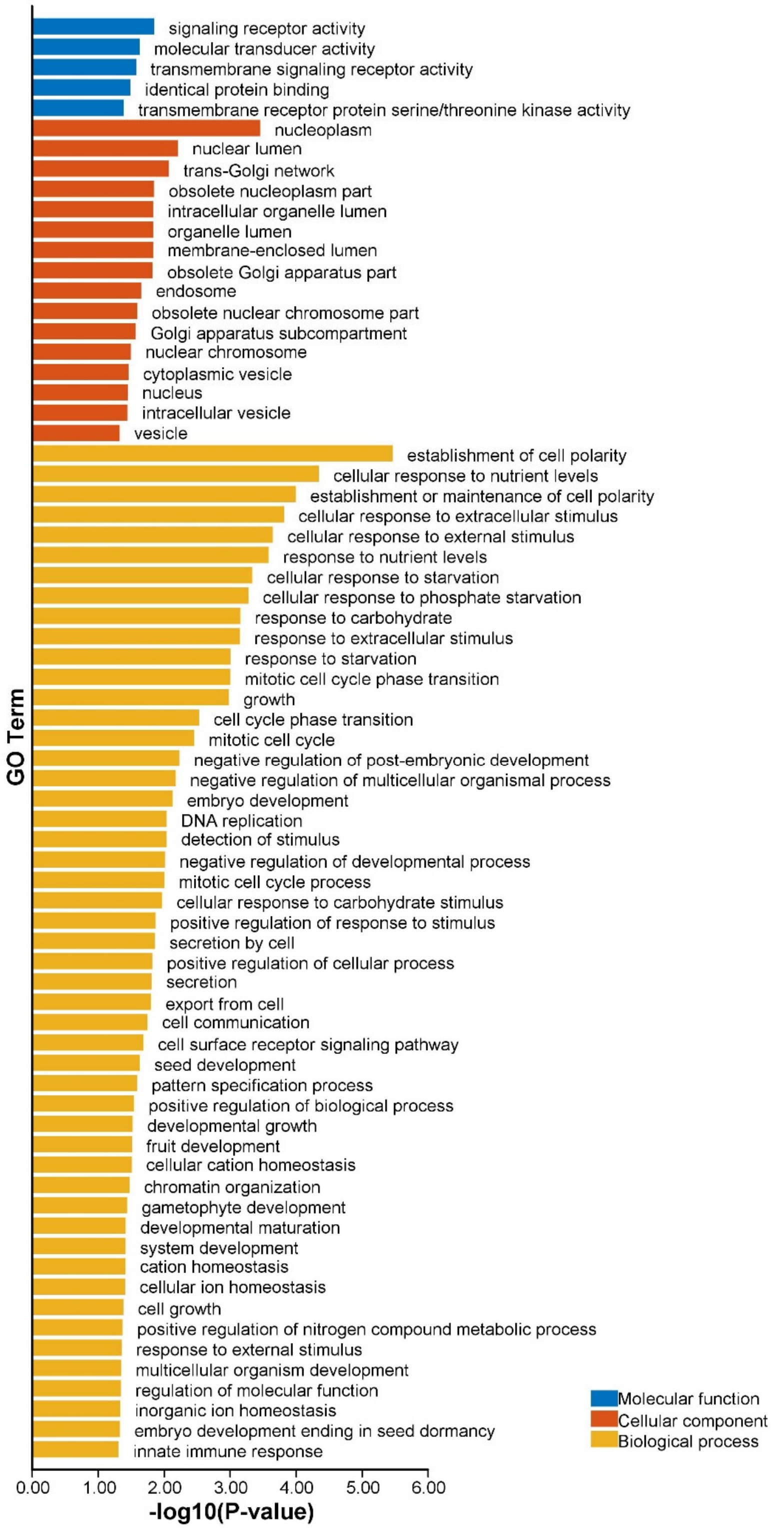

Figure S7. GO analysis of putative mmi-miR165/166 target genes.

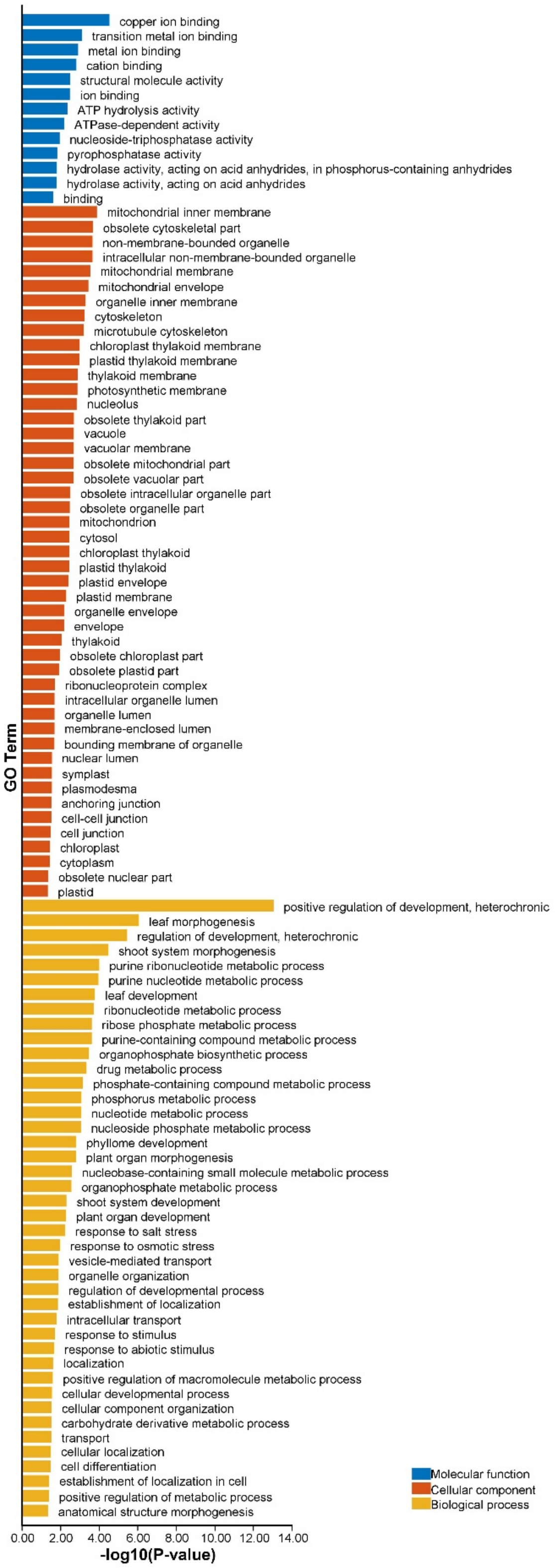

Figure S8. GO analysis of putative mmi-miR319 target genes.

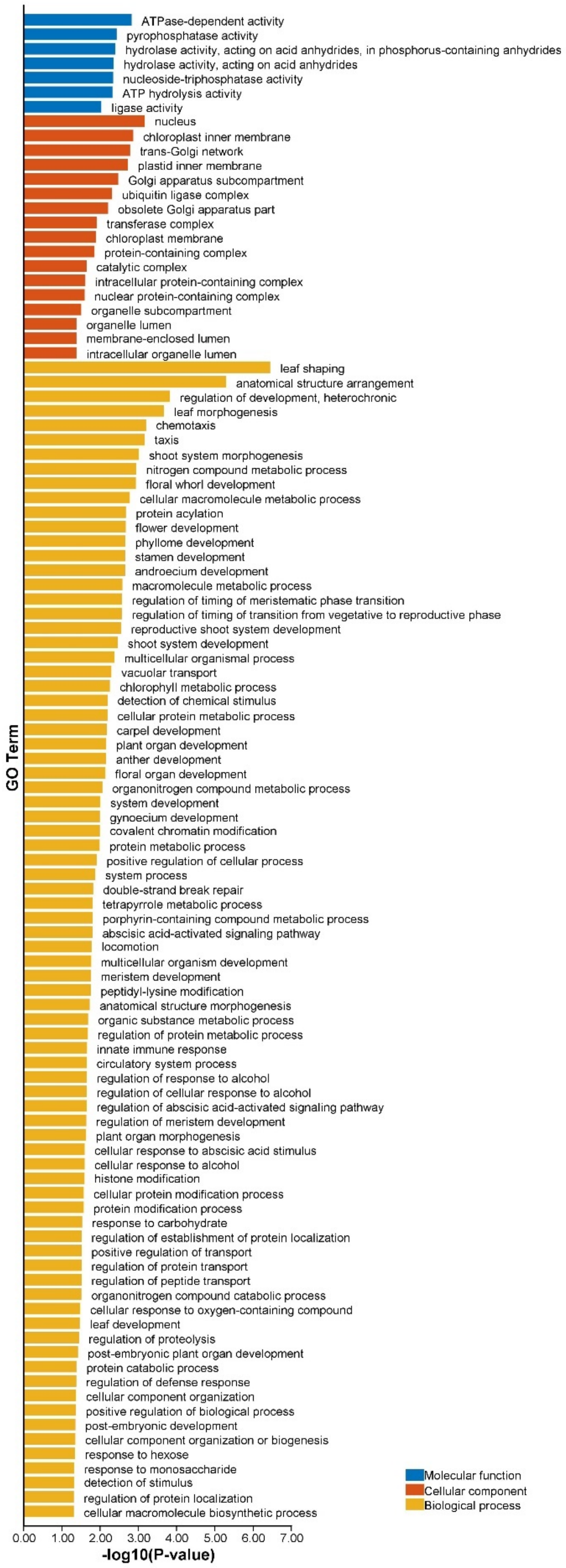

Figure S9. GO analysis of putative mmi-miR156 target genes.
